# Deciphering the Interrelationship of *arnT* Involved in Lipid-A Alteration with the Virulence of *Salmonella* Typhimurium

**DOI:** 10.3390/ijms25052760

**Published:** 2024-02-27

**Authors:** Chandran Sivasankar, Khristine Kaith Sison Lloren, John Hwa Lee

**Affiliations:** College of Veterinary Medicine, Jeonbuk National University, Iksan 54596, Republic of Korea

**Keywords:** *arnT*, gene deletion, intracellular survival, biofilm, swarming, in vivo colonization

## Abstract

The lipopolysaccharide (LPS) that resides on the outermost surface and protects Gram-negative bacteria from host defenses is one of the key components leading to Salmonella infection, particularly the endotoxic lipid A domain of LPS. Lipid A modifications have been associated with several genes such as the *arnT* that encodes 4-amino-4-deoxy-L-arabinose transferase, which can be critical for bacteria to resist cationic antimicrobial peptides and interfere with host immune recognition. However, the association of *arnT* with virulence is not completely understood. Thus, this study aimed to elucidate the interrelationship of the major lipid A modification gene *arnT* with *Salmonella* Typhimurium virulence. We observed that the *arnT*-deficient *S.* Typhimurium (JOL2943), compared to the wild type (JOL401), displayed a significant decrease in several virulence phenotypes such as polymyxin B resistance, intracellular survival, swarming, and biofilm and extracellular polymeric substance (EPS) production. Interestingly, the cell-surface hydrophobicity, adhesion, and invasion characteristics remained unaffected. Additionally, LPS isolated from the mutant induced notably lower levels of endotoxicity-related cytokines in RAW and Hela cells and mice, particularly IL-1β with a nine-fold decrease, than WT. In terms of in vivo colonization, JOL2943 showed diminished presence in internal organs such as the spleen and liver by more than 60%, while ileal infectivity remained similar to JOL401. Overall, the *arnT* deletion rendered the strain less virulent, with low endotoxicity, maintained gut infectivity, and reduced colonization in internal organs. With these ideal characteristics, it can be further explored as a potential attenuated *Salmonella* strain for therapeutics or vaccine delivery systems.

## 1. Introduction

*Salmonella enterica* is responsible for a range of illnesses in both animals and humans, encompassing mild to severe infections with the potential for fatality. Various strains of *Salmonella* Typhimurium induce enteric infections in humans and severe infections in a variety of animal species [1,2]. The ability of *Salmonella* to infect diverse hosts, exhibit systemic and multi-organ infectivity, and undergo virulence attenuations has positioned it as a promising platform for vaccine and therapeutic delivery. In the past years, numerous studies on the potential use of *Salmonella* for prophylactics and therapeutics have been documented, leading to the development of *Salmonella*-based therapeutic and prophylactic products [3,4,5,6,7]. However, with various methods to attenuate *Salmonella* for such purposes, endotoxicity is one of the major obstacles to the implementation of *Salmonella* for disease prevention and therapy which may cause inflammation in multiple organs [8,9]. 

The lipid-A part of LPS is the key component of endotoxicity, which interacts with myeloid differentiation-2 (MD-2) and Toll-like Receptor-4 (TLR-4) and activates the cascade of cytokine storms and inflammation [10]. The complete removal of lipopolysaccharide (LPS) or lipid-A to remove the unwanted effect of endotoxicity is not possible as it is indispensable for outer membrane integrity. Thus, the endotoxicity can be reduced by altering the lipid-A functional groups [11].

Multiple genes are involved in the alteration of hydrophobic and hydrophilic moieties of lipid A [12]. The modification of the number of acyl groups, change in the length of the acyl group, and charge modifications are major strategies [8]. These lipid-A modifications help the bacteria evade the host immune system [13]. The gene *arnT* encodes 4-amino-4-deoxy-L-arabinose transferase, which alters the charge of lipid A by adding a positively charged amino-arabinose residue. It primarily confers resistance to cationic antimicrobial peptide (CAMP) resistance or polymyxin B resistance [14]. However, the association of *arnT* with virulence is not completely understood. Thus, understanding its role in pathogenesis and its interlink with multiple virulence factors will be highly useful in developing prophylactic and therapeutic *Salmonella* strains without the peril of endotoxicity. In this prospect, the present investigation focused on studying the impact of *arnT* deletion in *S*. Typhimurium on multiple virulence factors such as initial infection and colonization, starvation sustainability, swarming, biofilm and associated phenotypes, host colonization, and survival.

## 2. Results

### 2.1. Effects of arnT Deletion on Polymyxin B Sensitivity, Adhesion, Invasion, Intracellular Survival, and Starvation 

JOL2943 was constructed using the λ-Red gene deletion method. The developed mutant was confirmed through inner primer PCR, in which no band was detected specific to the *arnT* gene, whereas, in JOL401, a band was detected at 250 bp (Figure S1). In addition, flanking primer PCR confirmation was performed, in which a band at 1.6 kb was detected in JOL401, but in the mutant, the band was observed only at 600 bp, confirming the deletion of the *arnT* gene (Figure S2).

**Figure 1:**
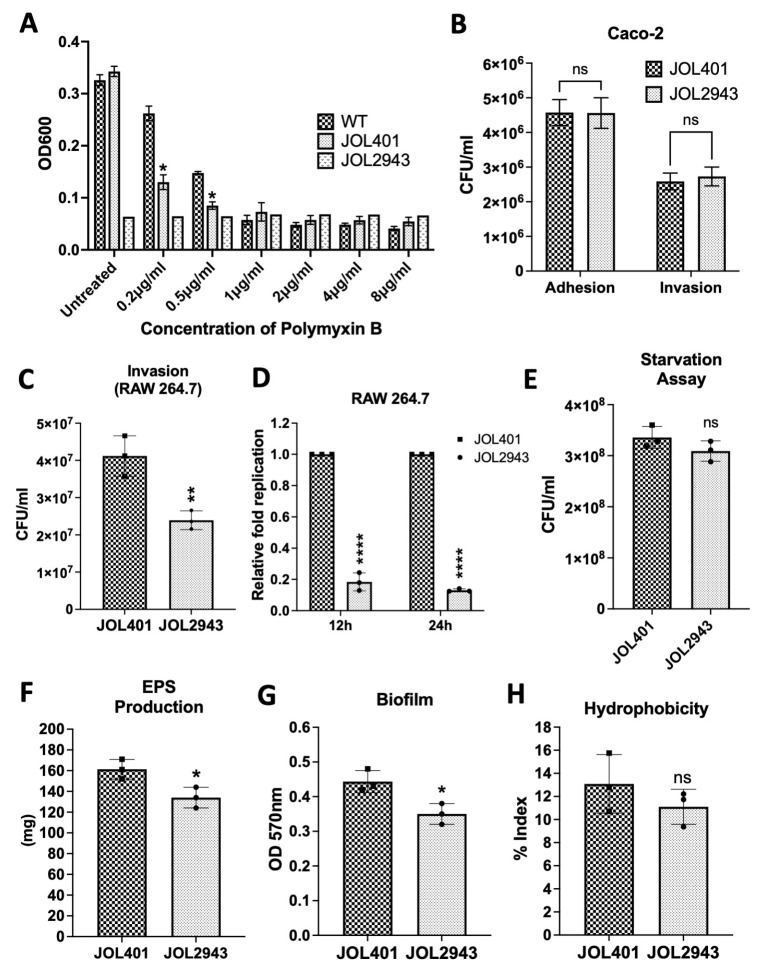
Virulence phenotypes of wild-type ST and *arnT*-deficient ST. (**A**) Bar diagram of polymyxin B sensitivity; bar heights indicate the growth of bacterial culture as optical density at 600 nm. (**B**) Adhesion and invasion in Caco-2 cells. (**C**) Invasion in RAW cells. (**D**) Relative fold intracellular replication or survival in RAW cells of JOL2943 and JOL401; Y-axis indicates fold intracellular replication expressed as CFUs recovered at 12 and 24 hpi relative to those at 2 hpi (invasion). (**E**) Bar diagram of starvation sustainability; Y-axis indicates the number of viable cells. (**F**) Amount of extrapolymeric substance (EPS) production. (**G**) Level of biofilm development by JOL2943 and JOL401. (**H**) Level of cell surface hydrophobicity of JOL2943 and JOL401 represented by the hydrophobicity index. The data presented are from at least three independent experiments performed in duplicates. Means were compared using *t*-test and two-way ANOVA. ^ns^ *p* > 0.05, * *p* < 0.05, ** *p* < 0.01, **** *p* < 0.0001.

The susceptibility to cationic antimicrobial peptides was first tested using polymyxin B. A higher sensitivity was observed in JOL2943 compared to JOL401 at a concentration of 0.5 and 0.2 µg/mL (Figure 1A). The ability of the JOL2943 strain to adhere to and invade Caco-2 cells was then compared with JOL401. JOL2943 showed similar adherence and invasion potential compared to JOL401 (Figure 1B). But the invasion in RAW macrophage cells was significantly reduced by up to 22% (Figure 1C). For intracellular survival, JOL2943 had a mean fold value of 0.18 at 12 h and 0.13 at 24 h relative to the WT, indicating a significant decrease in the intracellular replication of JOL2943 in macrophages (Figure 1D). Additionally, the survivability of JOL2943 in nutrient-less condition was assessed using a starvation assay in M9 media. The results indicated that there was no significant difference observed in the survival assay (Figure 1E).

### 2.2. Effects of arnT Deletion on Biofilm, EPS Production, and Swarming

**Figure 2:**
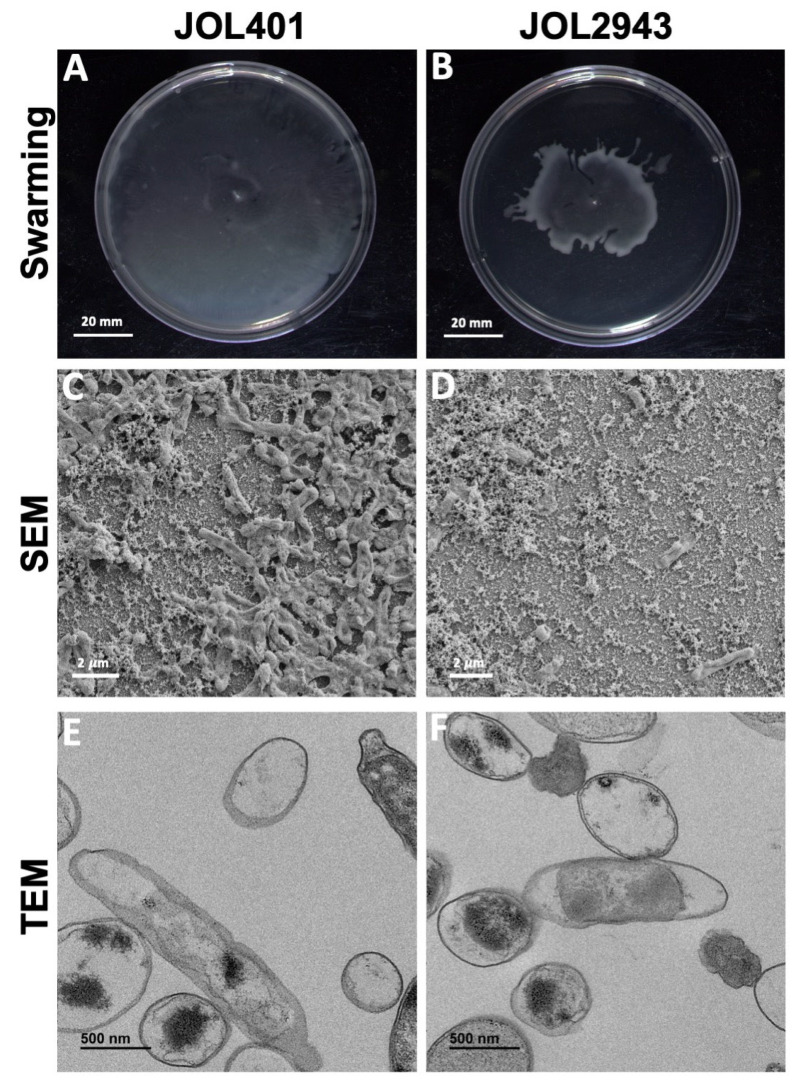
Swarming motility and electron microscopic images. (**A**,**B**) The swarming motility assay was performed for JOL2943 and JOL401 in 0.5% LB agar with 0.5% glucose and incubated for 9 h. (**C**,**D**) Scanning electron microscopic imaging of biofilm by JOL2943 and JOL401 was performed at a magnification of 5000×. (**E**,**F**) Transmission electron microscopic imaging was performed at a magnification of 30,000×.

The ability of biofilm development was assessed by the quantitative biofilm assay in 12-well plates. JOL2943 exhibited significantly less biofilm of 25% than JOL401 (Figure 1G). The adhered biofilms were also examined through SEM analysis, indicating that the wild-type cells were clumped together with EPS, whereas in JOL2943, cells were separated and not completely enclosed by EPS as shown in the scanning electron microscopy (SEM) in Figure 2C,D. The TEM results also revealed that JOL2943 showed less cell-bound EPS (Figure 2E,F). The EPS quantification result showed that JOL2943 produced 17% less EPS than JOL401 (Figure 1F). At the same time, the cell surface hydrophobicity was not significantly changed in JOL2943 (Figure 1H). Moreover, the effect of *arnT* deletion on motility was assessed through a swarming assay. After the incubation period, JOL401 swarmed until the boundary of the well plate, whereas JOL2943 was found to be considerably less motile than JOL401 (Figure 2A,B).

### 2.3. Assessment of Endotoxicity and Host Cytokine Responses

**Figure 3:**
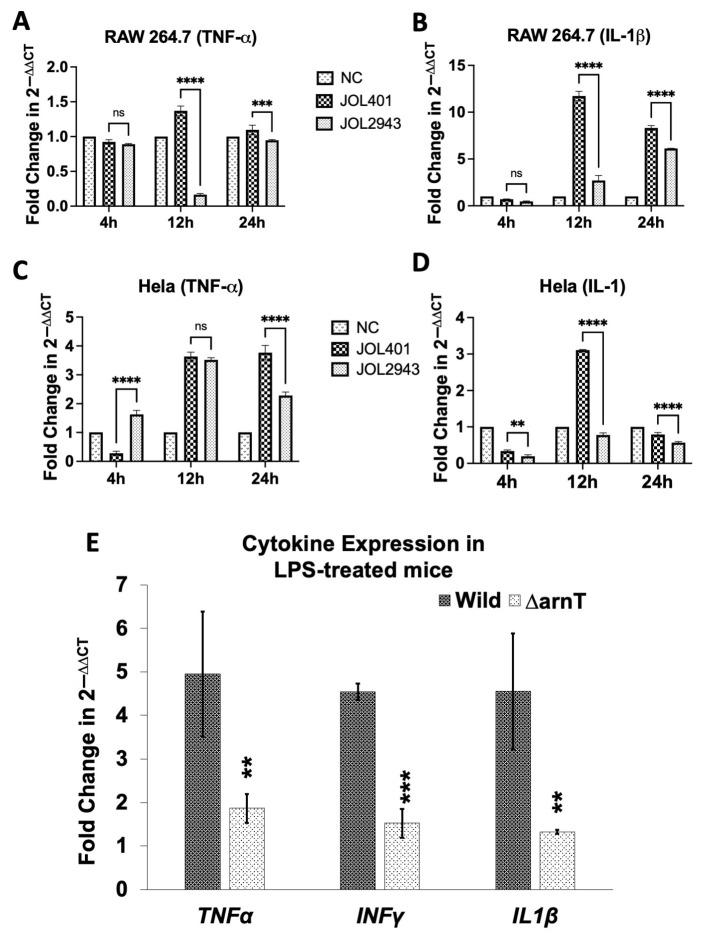
Changes in the expression level of endotoxicity-related cytokines such as IFN-γ, TNF-α, and IL-1β. Level of pro-inflammatory cytokines, (**A**) TNF-α and (**B**) IL-1β, induced by LPS from JOL2943 and JOL401 in RAW cells at 4, 12, and 24 hpi. Level of cytokines (**C**) TNF-α and (**D**) IL-1, induced by LPS from JOL2943 and JOL401 in Hela cells at 4, 12, and 24 hpi. (**E**) Level of pro-inflammatory cytokines in LPS-treated mice. The expression is plotted as mean fold changes (2^−ΔΔCT^). The data presented are from at least three independent experiments performed in duplicates. Means of JOL401 and JOL2943 were compared through two-way ANOVA. ^ns^ *p* > 0.05, ** *p* < 0.01, *** *p* < 0.001, **** *p* < 0.0001.

The induction of endotoxicity was assessed by measuring the mRNA expression level of endotoxicity-related cytokines such as TNF-α and IL-1β in isolated LPS-treated RAW and Hela cells and mice spleen. The cytokine expression of TNF-α and IL-1β was significantly reduced at 12 h and 24 h post infection of cells treated with LPS isolated from JOL2943 (Figure 3A,B), whereas in Hela cells, LPS isolated from JOL2943 induced significantly low TNF-α at 24 hpi and IL-1 at 12 h and 24 hpi (Figure 3C,D). In the in vivo experiment, the cytokine level was reduced by up to 2.6 to 3.4 folds in mice inoculated with LPS isolated from JOL2943 (Figure 3E). Overall, the qPCR results indicated that *arnT* deletion strongly reduced the endotoxicity in murine and human cell lines and in mice.

### 2.4. Serum Susceptibility Assay

**Figure 4:**
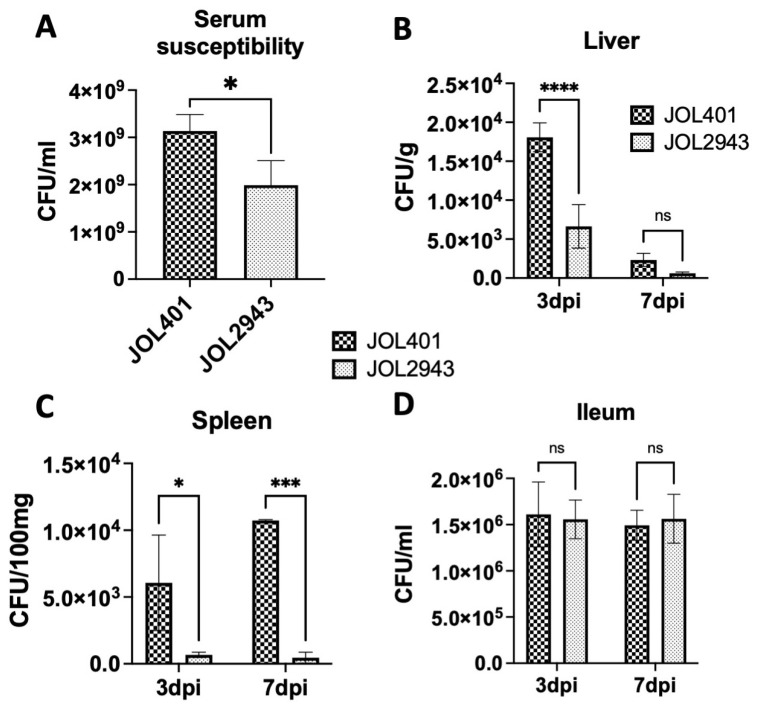
Serum sensitivity and bacterial load in different organs of mice. (**A**) Bar diagram of serum sensitivity; bar heights indicate the viability of bacterial cultures; data were compared using Student’s *t*-test. Microbial load of JOL401 and JOL2943 strains in the (**B**) liver, (**C**) spleen, and (**D**) ileum on days 3 and 7 post infection via oral inoculation with 10^9^ CFU. The data presented are from at least three independent experiments performed in duplicates. Means were compared using two-way ANOVA. ^ns^ *p* > 0.05, * *p* < 0.05, *** *p* < 0.001, **** *p* < 0.0001.

The serum sensitivity of JOL2943 was assessed by incubating the *Salmonella* cells with mice serum. After treatment with serum, the viability of JOL2943 was reduced to 30% relative to the wild-type strain (Figure 4A), indicating the sensitivity of JOL2943 to antimicrobial peptides present in the serum.

### 2.5. In Vivo Host Colonization

The infectivity and organ-colonization ability of JOL2943 were assessed by comparing them with JOL401. The colonization of JOL2943 in mice liver (Figure 4B) was significantly reduced to 63% and 73% at 3 dpi and 7 dpi, respectively. In the spleen, the bacterial load in the ST wild-type infected mice was increased at 3 dpi and 7 dpi, while the bacterial load of JOL2943 infected mice was significantly reduced by 88% and 95% at 3 dpi and 7 dpi, respectively (Figure 4C). However, the bacterial load in the ileum did not vary significantly, which implied that the *arnT* deletion may affect the invasion and infection in the liver and spleen but not the initial infection in the ileum (Figure 4D).

## 3. Discussion

Lipopolysaccharide (LPS) serves as a constituent of *Salmonella*’s outer membrane, featuring lipid A as a crucial element recognized by Toll-like receptor 4 (TLR4) and MD-2 on innate immune cells in the host. The interaction between LPS and TLR4 triggers the activation of the transcription factor NFκB, consequently inducing the synthesis of pro-inflammatory cytokines. This process initiates both the adaptive immune response and incites endotoxicity and inflammation [10]. *Salmonella* has the capability to modify its lipid A molecule, leading to a substantial modification of TLR4 signaling towards NFκB. Consequently, this alteration of lipid A serves as a pivotal immune evasion strategy [15]. Lipid A is an amphipathic molecule; the alteration of the acyl group number or length and the modification of the charge by adding charged moieties are the major lipid A alteration mechanisms [10]. Several genes or orthologs of PhoPQ, PmrAB, and RcsCDB have been shown to regulate LPS modification genes [12]. One such example is *arnT*, encoding 4-amino-4-deoxy-L-arabinose (L-Ara4N) transferase, a lipid A modifying enzyme that adds a positively charged amino-arabinose group, thus altering the net charge of lipid A and conferring CAMP or polymyxin resistance [16]. In addition, according to previous studies, the alteration of the net charge of lipid A also affects TLR4 interaction and consequently modifies the expression of pro-inflammatory cytokines [17]. Although several studies have demonstrated the role of ArnT as an integral membrane protein for transferring precursors to lipid A, the influence of *arnT* on virulence and pathogenesis has not yet been well studied. Therefore, in the present study, we investigated the possible association of *arnT* on bacterial physiology and virulence in vitro and in vivo of *Salmonella* Typhimurium to further understand its possible contribution to bacterial virulence.

Firstly, JOL2943 exhibited significantly higher susceptibility to polymyxin B, even at low concentrations compared to JOL401. Polymyxin B is an anionic antibiotic that can electrostatically interact with lipid A and make pores in the outer membrane. If the net positive charge of lipid A becomes low, the outer membrane fails to repel the polymyxin B molecule and becomes more susceptible [18]. Thus, the results imply that the removal of amino-arabinose from lipid A due to *arnT* deletion altered the overall charge of the molecule and, thus, increased the susceptibility to polymyxin B. This has also been similarly observed in other covalent modifications of phosphate groups in lipid A which confer resistance to polymyxin B and aminoglycosides and cationic antimicrobial peptides, which can also be associated with bacterial virulence [13]. 

The effect of *arnT* deletion in the infection process of *S.* Typhimurium was assessed in vitro by determining the level of adhesion and invasion in non-phagocytic Caco-2 cells. The *arnT*-deficient *S.* Typhimurium still retained its adhesion and invasion ability similar to JOL401, suggesting that SPI-1-related phenotypes which are associated with *Salmonella* invasion [19] may not be significantly affected. However, the intracellular viability of the mutant in phagocytic RAW cells was significantly reduced, potentially indicating the effect of *arnT* deletion on SPI-2 genes that are primarily responsible for the survival of *Salmonella* within the host cell [20]. Altogether, the results revealed that the deletion of *arnT* affected SPI-2-related intracellular survival but not SPI-1 phenotypes [21]. These results corroborated other reports of Lipid A modifications in *Francisella tularensis* and *Burkholderia pseudomallei*, which also resulted in reduced intracellular survival [22,23], indicating the potential effect of *arnT* deletion on the SPI-2 genes associated with survival in host cells. 

LPS modifications in *E. coli* have been reported to be connected with starvation survival [24]. Therefore, JOL2943 was further tested for starvation survivability but no significant changes were observed, which implies that the gene may have had no role in the starvation assay. In contrast, JOL2943 showed a significant reduction in EPS production and biofilm formation. The results are in connection with previous reports signifying the impact of LPS’s structural modifications on biofilm development [25,26,27]. The EPS production and biofilm are interconnected phenotypes, and both were parallelly reduced in JOL2943. The reduction of the biofilm was further confirmed through scanning electron microscopic analysis and transmission electron microscopic analysis, which showed less cell-bound EPS. However, the cell surface hydrophobicity (CSH) was not affected. Therefore, the biofilm reduction of JOL2943 was independent of CSH. Relatively, a previous study on *pagL* deletion, another lipid A-modifying enzyme, has also resulted in reduced biofilm development in *S*. Typhimurium [28], implying that modifications in Lipid A affect biofilm formation. Moreover, JOL2943 showed less swarming motility than JOL401. Both the swarming and biofilm are quorum-sensing dependent virulence factors [29]. In our observation, both of these two virulence factors were not completely arrested but were reduced at a level of 25%. These results additionally support previous reports on the impact of deleting LPS-related genes on swarming in other Gram-negative bacteria [30,31].

As modifications in lipid A could affect the recognition by Toll-like receptor 4 (TLR4)-MD2, a pattern recognition receptor (PRR) of the mammalian innate immune system [13], the signaling cascade leading to inflammation and cytokine production can be changed. In this study, we further investigated the effect of *arnT* deletion in the expression of cytokines such as TNF-α and IL-1β in vitro in Hela and RAW cells as well as in vivo in the LPS-treated mice. These cytokines are produced by the host cell in response to LPS and are also involved in endotoxicity [32]. The results revealed a significant decrease in cytokine expression both in vitro and in vivo by JOL2943 compared to JOL401, indicative of the impact of lipid A modifications on triggering signaling pathways for host immune response including reduced endotoxicity. It is conjectured that the modified lipid A in JOL2943 may activate the TLR4-TRAM-TRIF pathway, triggering type I interferon, leading to immune activation, as opposed to the TLR4-TIRAP-MyD88 pathway that induces inflammatory cytokines [10,33], although this needs further validation.

Modifications to lipid A via *arnT* deletion may also mediate serum susceptibility phenotype and virulence in vivo. JOL2943 was significantly more susceptible to mouse serum than JOL401, which indicates that the mutant is more sensitive to CAMPs present in serum. This result can also be correlated with the polymyxin B sensitivity of the mutant as well as previous reports [34]. These findings are further supported by the decreased persistence of the *arnT*-deficient *S.* Typhimurium in vivo, wherein the bacterial colonization of JOL2943 in the liver and spleen was significantly reduced compared to JOL401. However, although the bacterial load was significantly reduced in these organs, the infectivity in the ileum of the mutant remained similar to that of JOL401, indicating that the adhesion and invasion abilities of the mutant are not affected, which correlated with the in vitro study. Overall, the in vivo results indicate that the initial infection in the gut by *arnT* was not impaired, but the systemic infection was considerably reduced. 

Taken together, this study demonstrates that *arnT* deletion in *S.* Typhimurium, which modifies lipid A, was highly sensitive to polymyxin B and serum CAMPs. Although adhesion and invasion were not affected, the intracellular survival of the *arnT*-deficient *Salmonella* in macrophages was significantly reduced. In addition, the mutant exhibited reduced biofilm, EPS production, and swarming motility, while the cell surface charge and starvation survival were not altered. The absence of *arnT* not only reduced the endotoxicity-related cytokines but also decreased the virulence as shown by reduced bacterial load in the organs of infected mice. Overall, the present investigation sheds light and further understanding on the interconnections of the *arnT* gene with the bacterial pathophysiology and virulence of *Salmonella* Typhimurium. With the ideal characteristics such as reduced endotoxicity and virulence but retained adhesion and invasion of JOL2943 demonstrated in this study, the *arnT*-deficient *S.* Typhimurium or in combination with other attenuating modifications can be further explored for optimizing *Salmonella* strains for bacterial vaccine delivery and therapeutics.

## 4. Materials and Methods

### 4.1. Animal Ethics Statement

All experiments involving animals were conducted in accordance with the authorization granted by the Jeonbuk National University Animal Ethics Committee (IACUC, NON2022-024-002) and adhered to the regulations outlined in the Korean Animal Protection Law of 2007, specifically Article 13. Female BALB/c mice, aged six weeks and free from specific pathogens, were procured from Samtako (Osan, Republic of Korea). The mice were housed in a designated animal facility, receiving unrestricted access to antibiotic-free food and water. 

### 4.2. Bacterial Strains and Growth Conditions, Plasmids, Primers

The bacterial strains, plasmids, and PCR primers employed in this investigation are outlined in Table 1. The cultivation of *Salmonella* Typhimurium JOL401 and the mutant strain featuring the deletion of the *arnT* gene was performed in Luria Bertani (LB; BD, Sparks, NV, USA) medium at 37 °C. 

### 4.3. Gene Deletion

The construction of the mutant strain followed a previously documented standard lambda Red recombination protocol [38]. Briefly, the electroporation of JOL401 with the helper plasmid pKD46 preceded the deletion of the target gene from the host strain. The pKD46 plasmid supplied the necessary inducible Red lambda components for recombination. Subsequently, the target gene *arnT* was substituted with the *catR* gene derived from a linear PCR product amplified from the pkD3 plasmid. Recombinant clones were identified through plating on LB agar supplemented with 25 μg/mL chloramphenicol. Confirmation of successful *arnT* gene deletion was achieved using inner primers. The *catR* gene within the resultant mutant was excised using the pCP20 plasmid, which encodes flippase. Verification of the mutant was conducted through flanking PCR primers (Table 1). Three confirmed clones of ∆*arnT* mutants were used in the succeeding assays. 

### 4.4. Polymyxin B Sensitivity

The evaluation of the response of JOL2943 to polymyxin B was conducted through a broth microdilution assay with slight modifications [39,40]. Various concentrations of polymyxin B, spanning 0.2 to 8 μg/mL, were employed in the experiment. A total of 10^6^ bacteria were inoculated in each well. Briefly, a bacterial suspension with an optical density (OD) of 0.63, corresponding to approximately a concentration of 5 × 10^8^ cells/mL, was prepared. This suspension was diluted at a 1:5 ratio to achieve a working suspension with a concentration of approx. concentration of 10^8^ cells/mL. Subsequently, 2 μL of the working suspension was added to each well, and the total volume in each well was adjusted to 200 μL. Following an overnight incubation period, the optical density at 600 nm was measured using a multi-well plate reader (Tecan, Männedorf, Switzerland). 

### 4.5. Adhesion, Invasion, and Intracellular Survival Assay

The evaluation of adhesion, invasion, and intracellular survival of the *Salmonella* JOL2943 involved the use of Caco-2 and RAW cells. In brief, a fully formed monolayer was maintained in a 24-well cell culture plate, and infection was carried out with JOL401 and JOL2943 *Salmonella* strains at a multiplicity of infection (MOI) of 40. For the adhesion assay, cells were subjected to a 30 min incubation, followed by three washes with PBS and lysis with 0.1% Triton X-100 for 10 min. Enumeration of bacterial cells was performed by plating on BGA plates using 10-fold serial dilutions.

In the invasion assay, *Salmonella* strains infected the cell monolayer for 2 h. After three PBS washes, the cells were incubated with complete Dulbecco’s modified Eagle medium (DMEM) containing gentamicin (100 μg/mL) for 1.5 h to eliminate extracellular bacteria. Following three PBS washes, the monolayers were treated with 0.1% Triton X-100 for 10 min, and bacterial counts were determined on BGA plates.

For assessing the intracellular survival of *Salmonella* JOL401 and mutant strains, RAW cells were infected with JOL401 and JOL2943 *Salmonella* strains for 2 h. After three washes, the cells were treated with DMEM containing gentamicin (100 μg/mL) and further incubated for 12 h. Subsequently, cell lysis was carried out with 0.1% Triton X-100 for 10 min, and viable bacterial cells were enumerated through plating on BGA plates. 

### 4.6. Starvation Assay

Freshly grown 8 h cultures of *S*. Typhimurium JOL401 and JOL2943 were taken and diluted to 0.5 OD at 600 nm using sterile M9 buffer. The diluted bacterial suspension was incubated at 37 °C for 72 h. After incubation, the bacterial suspension was serially diluted and plated on LB agar. The CFU was calculated by manually counting the number of colonies [41].

### 4.7. EPS Production Assay

*S*. Typhimurium JOL401 and JOL2943 were cultured in 100 mL volumes at 37 °C for 24 h. Following incubation, cells and cell-free culture supernatant (CFCS) components were separated through centrifugation at 8228× *g* for 10 min. The resultant cell pellet underwent a sterile phosphate-buffered saline (PBS) wash to eliminate any remaining CFCS. Subsequently, the cells were resuspended in a 100 mL isotonic buffer (10 mM Tris/HCl pH 8.0, 10 mM EDTA, 2.5% NaCl) and incubated at 4 °C overnight. Following this incubation, the cell suspension underwent a 5 min vortexing session, followed by centrifugation at 8228× *g* for 10 min. The supernatant, which contained cell-bound exopolysaccharides (EPS), was combined with CFCS. A mixture of ice-cold ethanol at a 1:3 ratio was added to the combined supernatants, and the solution was incubated at −20 °C overnight. Subsequently, EPS was isolated through centrifugation at 18,514× *g* for 30 min at 4 °C. The resulting pellet was washed with 70% ethanol and weighed after complete drying.

### 4.8. Biofilm Assay

JOL401 and JOL2943 were cultured in a 24-well plate to assess the biofilm development. The TSB broth in individual wells was inoculated with 1% of bacterial cell suspension with a cell density of 1 × 10^8^ CFU/mL, and then the well plate was incubated at 37 °C for 24 h. After incubation, the liquid culture comprising planktonic cells was carefully eliminated, and the adhered biofilm was washed with sterile PBS. Then, the biofilm was stained with 0.4% crystal violet for 2 min, and the biofilm was again washed twice with water. Finally, the biofilm was de-stained with 70% ethanol for 1 h and read spectrophotometrically at 570 nm.

### 4.9. SEM and TEM Microscopic Visualization

For scanning electron microscopy (SEM), coverslips were positioned at the base of a 6-well plate, and biofilms were cultured in TSB medium for 24 h at 37 °C. Subsequently, the coverslips were fixed overnight at 40 °C with 2.5% glutaraldehyde in 0.1 M phosphate buffer and 0.1 M sucrose (pH 7.4) and processed for SEM analysis following a previously described protocol [42]. The samples were then coated with a gold–palladium alloy and examined using SEM (JSM-5200, JEOL, Tokyo, Japan).

To compare cell morphology and the presence of extracellular polymeric substances (EPS) in JOL401 and mutant *Salmonella* cells, transmission electron microscopy (TEM) was employed. Both JOL2943 and the wild-type JOL401 strains were cultured to the mid-log phase (OD600 of 0.6). Bacterial cells were harvested by centrifugation and fixed in a solution of 2% paraformaldehyde and 2% glutaraldehyde in 0.05 M sodium cacodylate (pH 7.2) overnight at 4 °C. Following three washes with 0.05 M sodium cacodylate (pH 7.2), the specimens were post-fixed with 1% osmium tetraoxide in 0.05 M sodium cacodylate (pH 7.2) for 1.5 h at 4 °C. After distilled water rinses, en bloc staining was performed with 0.5% uranyl acetate for 30 min at 4 °C. Sequential dehydration was carried out using an ethanol gradient of 30%, 40%, 50%, 70%, 80%, 90%, and 3 × 100%. Following a transition with 100% propylene oxide, the cell pellet was infiltrated with a resin mixture and polymerized at 60 °C for 48 h. Sectioning was performed using an ultramicrotome, and observations were made with TEM (Hitachi, Tokyo, Japan) at an acceleration voltage of 100 kV and magnification of 30,000×.

### 4.10. MATH Assay

The impact of *arnT* deletion on the cell surface hydrophobicity (CSH) of *S.* Typhimurium was assessed using the MATH assay [43]. Sterile PBS was utilized to prepare cell suspensions for both JOL401 and mutant strains. In a 3 mL cell suspension (0.5 OD), an equivalent volume of toluene (Merck Ltd., Seoul, Republic of Korea) was introduced, and the mixture was vortexed for 60 s. Subsequent to vortexing, the tubes were left undisturbed until the distinct phases were separated, and the toluene phase was then removed. Any residual solvent traces were eliminated by allowing the aqueous phase to incubate at room temperature overnight. The OD600 of the aqueous phase was measured using a Shimadzu UV–Visible spectrometer (UV 2450, Kyoto, Japan), and the CSH was determined as a hydrophobic index (HI) using the formula HI = [1 − (OD600 after vortexing/OD600 before vortexing)] × 100.

### 4.11. Swarming Assay

The motility assay was performed using JOL401 and JOL2943 bacterial suspensions with approximately 1 × 10^8^ CFU/mL. One microliter of bacterial suspensions was softly spotted in the middle of the LB swarming agar plates with 0.5% agar and 0.5% glucose [*w*/*v*] and incubated at 37 °C. The distance migrated by bacterial cultures was compared after 9 h post inoculation.

### 4.12. RT-qPCR Analysis of Cytokine Expression In Vitro and In Vivo

The lipopolysaccharide was isolated from ST JOL401 and JOL2943 strains using an LPS extraction kit, following the manufacturer’s protocol (iNtRON Biotechnology, Seoul, Republic of Korea). The Raw and Hela cells were then treated with 100 ng/mL of isolated LPS. Similarly, mice (*n* = 6) were treated with 100 ng/mL of isolated LPS intramuscularly. The cells treated with lipopolysaccharide (LPS) and the spleens of mice subjected to LPS treatment were harvested, and the RNA was isolated using the Hybrid R kit (GeneAll, Seoul, Republic of Korea) following the manufacturer’s protocol. Subsequently, cDNA was synthesized using a reverse transcriptase kit (ELPISBIO, Daejeon, Republic of Korea). Real-time quantitative PCR (RT-qPCR) was employed to assess the expression levels of cytokines TNF-α and IL-1β. The relative gene expression was measured in triplicate using SYBR Green/ROX master mix in an ABI StepOnePlus Real-Time PCR system with reaction conditions of 95 °C for pre-incubation followed by 40 cycles of 30 s each at 95 °C, 55 °C, and 72 °C. The relative expression was determined with the 2^−ΔΔCT^ method [44] using the unstimulated cells as the reference sample and GAPDH as the endogenous control.

### 4.13. Serum Susceptibility Assay

The sensitivity of JOL2943 to serum was tested via a serum susceptibility test using a previously described method with modifications [45,46,47]. Briefly, blood was collected from mice and incubated for at least an hour before serum was isolated through centrifugation at 2000 rpm for 20 min. JOL401 and JOL2943 cell suspensions at a CFU of 4 × 10^9^/mL were mixed with freshly prepared mice serum and incubated for 3 h at 37 °C. After incubation, the serum and cell suspension mixtures were serially diluted and spread on LB agar for counting viable cells. 

### 4.14. In Vivo Host Colonization

The in vivo colonization of JOL2943 was compared with JOL401. Female BALB/c mice aged six weeks (N = 12, *n* = 6) were evenly distributed to groups. Groups A and B were orally inoculated of either JOL401 or JOL2943 strains at a concentration of 10^9^ cells/100 μL. Daily monitoring of body weight and clinical signs continued until the termination of the experiment. On the third day and seventh day post inoculation, all mice in each group were humanely euthanized through cervical dislocation. Liver, spleen, and ileum samples were harvested, weighed, and homogenized in PBS. Subsequently, 100 μL of the homogenized samples was serially diluted and plated on BGA plates for colony-forming unit (CFU) enumeration.

### 4.15. Statistical Analysis

All data were analyzed using Student’s *t*-test and two-way ANOVA using Prism 10 (GraphPad Inc., San Diego, CA, USA) to compare the means among the groups and to compute the corresponding *p*-values. Data analyses were from at least three independent experiments performed in duplicates. Data in graphs are presented as the mean ± SEM with ^ns^ *p* > 0.05; * *p* < 0.05; ** *p* < 0.01; *** *p* < 0.001; **** *p* < 0.0001. 

## Figures and Tables

**Table 1:** Bacterial strains, plasmids, and primers used in this study.

Bacterial Strains/ Plasmids/Primers		Reference
**Bacterial Strains**		
JOL401	*Salmonella* Typhimurium wild-type	Lab stock
JOL2943	*Salmonella* Typhimurium ∆*arnT* mutant	This study
**Plasmids**		
pKD46	Ori101-repA101ts; encodes Lambda Red genes (exo, bet, gam); native terminator (tL3); arabinose-inducible for expression (ParaB); bla	[35]
pKD3	oriR6K gamma, bla (ampR), rgnB (Ter), catR, FRT	[35]
pCP20	Helper plasmid containing a temperature-inducible flp gene for removing the FRT-flanked chloramphenicol gene	[35]
**Gene Deletion** **primers**		
arnT pKD3	Forward— GAGCTGACCGCCAACGCTGAGCAGACTGGCAAGCACCAGAATGACGCCGAGTGTAGGCTGGAGCTGCTTC Reverse— ATCCCTGGCCGTGAAGGTTGGCTGGGGTGCCAACAGGCAGCGAGCGCCTCATGGGAATTAGCCATGGTCC	[35]
arnT inner	Forward—GCAACGCGGTACGTTTATCC Reverse—GAAACGCGCTATGCCGAAAT	[35]
arnT flanking	Forward—GAGCTGACCGCCAACGCTGA Reverse—ATCCCTGGCCGTGAAGGTTG	[35]
**qPCR cytokine** **primers**		
INF-γ	Forward—TCAAGTGGCATAGATGTGGAAGAA Reverse—TGGCTCTGCAGGATTTTCATG	[36]
TNF-α	Forward—CATCTTCTCAAAATTCGAGTGACAA Reverse—TGGGAGTAGACAAGGTACAACCC	[36]
IL-1β	Forward—TTCACCATGGAATCCGTGTC Reverse—GTCTTGGCCGAGGACTAAGG	[37]
GAPDH	Forward—TCACCACCATGGAGAAGGC Reverse—GCTAAGCAGTTGGTGGTGCA	[36]

## Data Availability

The data presented in the study are available on request from the corresponding author.

## Supplementary Materials

The supporting information can be downloaded at: https://www.mdpi.com/article/10.3390/ijms25052760/s1.

## References

[1] Jajere S.M. (2019). A review of *Salmonella enterica* with particular focus on the pathogenicity and virulence factors, host specificity and antimicrobial resistance including multidrug resistance. Vet. World.

[2] Rabsch W., Andrews H.L., Kingsley R.A., Prager R., Tschäpe H., Adams L.G., Bäumler A.J. (2002). *Salmonella enterica* Serotype Typhimurium and Its Host-Adapted Variants. Infect. Immun..

[3] Becerra-Báez E.I., Meza-Toledo S.E., Muñoz-López P., Flores-Martínez L.F., Fraga-Pérez K., Magaño-Bocanegra K.J., Juárez-Hernández U., Mateos-Chávez A.A., Luria-Pérez R. (2022). Recombinant Attenuated *Salmonella enterica* as a Delivery System of Heterologous Molecules in Cancer Therapy. Cancers.

[4] Hegazy W.A.H., Hensel M. (2012). *Salmonella enterica* as a Vaccine Carrier. Future Microbiol..

[5] Moreno M., Kramer M.G., Yim L., Chabalgoity J.A. (2010). *Salmonella* as Live Trojan Horse for Vaccine Development and Cancer Gene Therapy. Curr. Gene Ther..

[6] Chorobik P., Marcinkiewicz J. (2011). Therapeutic vaccines based on genetically modified *Salmonella*: A novel strategy in cancer immunotherapy. Pol. Arch. Med. Wewn..

[7] Broadway K.M., Scharf B.E. (2019). *Salmonella* Typhimurium as an Anticancer Therapy: Recent Advances and Perspectives. Curr. Clin. Microbiol. Rep..

[8] Kawasaki K. (2012). Complexity of lipopolysaccharide modifications in *Salmonella enterica*: Its effects on endotoxin activity, membrane permeability, and resistance to antimicrobial peptides. Food Res. Int..

[9] Kumar V. (2020). Toll-like receptors in sepsis-associated cytokine storm and their endogenous negative regulators as future immunomodulatory targets. Int. Immunopharmacol..

[10] Maeshima N., Fernandez R.C. (2013). Recognition of lipid A variants by the TLR4-MD-2 receptor complex. Front. Cell. Infect. Microbiol..

[11] Kong Q., Six D.A., Liu Q., Gu L., Wang S., Alamuri P., Raetz C.R.H., Curtiss R. (2012). Phosphate Groups of Lipid A Are Essential for *Salmonella enterica* Serovar Typhimurium Virulence and Affect Innate and Adaptive Immunity. Infect. Immun..

[12] Richards S.M., Strandberg K.L., Gunn J.S. (2010). *Salmonella*-Regulated Lipopolysaccharide Modifications. Subcell. Biochem..

[13] Needham B.D., Trent M.S. (2013). Fortifying the barrier: The impact of lipid A remodelling on bacterial pathogenesis. Nat. Rev. Microbiol..

[14] Zhou Z., Ribeiro A.A., Lin S., Cotter R.J., Miller S.I., Raetz C.R. (2001). Lipid A Modifications in Polymyxin-resistant *Salmonella* Typhimurium: PmrA-Dependent 4-Amino-4-Deoxy-L-Arabinose, and Phosphoethanolamine Incorporation. J. Biol. Chem..

[15] Matsuura M. (2013). Structural Modifications of Bacterial Lipopolysaccharide that Facilitate Gram-Negative Bacteria Evasion of Host Innate Immunity. Front. Immunol..

[16] Gunn J.S., Lim K.B., Krueger J., Kim K., Guo L., Hackett M., Miller S.I. (1998). PmrA–PmrB-regulated genes necessary for 4-aminoarabinose lipid A modification and polymyxin resistance. Mol. Microbiol..

[17] Kawasaki K., Ernst R.K., Miller S.I. (2005). Inhibition of *Salmonella enterica* Serovar Typhimurium Lipopolysaccharide Deacylation by Aminoarabinose Membrane Modification. J. Bacteriol..

[18] Moffat J.H., Harper M., Boyce J.D. (2019). Mechanisms of polymyxin resistance. Polymyxin Antibiotics: From Laboratory Bench to Bedside.

[19] Lou L., Zhang P., Piao R., Wang Y. (2019). *Salmonella* Pathogenicity Island 1 (SPI-1) and Its Complex Regulatory Network. Front. Cell. Infect. Microbiol..

[20] Fass E., Groisman E.A. (2009). Control of *Salmonella* pathogenicity island-2 gene expression. Curr. Opin. Microbiol..

[21] Hapfelmeier S., Stecher B., Barthel M., Kremer M., Müller A.J., Heikenwalder M., Stallmach T., Hensel M., Pfeffer K., Akira S. (2005). The *Salmonella* Pathogenicity Island (SPI)-2 and SPI-1 Type III Secretion Systems Allow *Salmonella* Serovar *typhimurium* to Trigger Colitis via MyD88-Dependent and MyD88-Independent Mechanisms. J. Immunol..

[22] Norris M.H., Somprasong N., Schweizer H.P., Tuanyok A. (2018). Lipid A Remodeling Is a Pathoadaptive Mechanism That Impacts Lipopolysaccharide Recognition and Intracellular Survival of *Burkholderia pseudomallei*. Infect. Immun..

[23] Yun J., Wang X., Zhang L., Li Y. (2017). Effects of lipid A acyltransferases on the pathogenesis of *F. novicida*. Microb. Pathog..

[24] Vogeleer P., Vincent A.T., Chekabab S.M., Charette S.J., Novikov A., Caroff M., Beaudry F., Jacques M., Harel J. (2019). *Escherichia coli* O157:H7 Responds to Phosphate Starvation by Modifying LPS Involved in Biofilm Formation. bioRxiv.

[25] Ciornei C.D., Novikov A., Beloin C., Fitting C., Caroff M., Ghigo J.-M., Cavaillon J.-M., Adib-Conquy M. (2010). Biofilm-forming Pseudomonas aeruginosa bacteria undergo lipopolysaccharide structural modifications and induce enhanced inflammatory cytokine response in human monocytes. Innate Immun..

[26] Mireles J.R., Toguchi A., Harshey R.M. (2001). *Salmonella enterica* Serovar Typhimurium Swarming Mutants with Altered Biofilm-Forming Abilities: Surfactin Inhibits Biofilm Formation. J. Bacteriol..

[27] Hathroubi S., Beaudry F., Provost C., Martelet L., Segura M., Gagnon C.A., Jacques M. (2016). Impact of *Actinobacillus pleuropneumoniae* biofilm mode of growth on the lipid A structures and stimulation of immune cells. Innate Immun..

[28] Hewawaduge C., Senevirathne A., Sivasankar C., Lee J.H. (2023). The impact of lipid A modification on biofilm and related pathophysiological phenotypes, endotoxicity, immunogenicity, and protection of *Salmonella* Typhimurium. Vet. Microbiol..

[29] Rana K., Nayak S.R., Bihary A., Sahoo A.K., Mohanty K.C., Palo S.K., Sahoo D., Pati S., Dash P. (2021). Association of quorum sensing and biofilm formation with *Salmonella* virulence: Story beyond gathering and cross-talk. Arch. Microbiol..

[30] Lee C., Mannaa M., Kim N., Kim J., Choi Y., Kim S.H., Jung B., Lee H.-H., Lee J., Seo Y.-S. (2019). Stress Tolerance and Virulence-Related Roles of Lipopolysaccharide in *Burkholderia glumae*. Plant Pathol. J..

[31] Bowden S.D., Hale N., Chung J.C.S., Hodgkinson J.T., Spring D.R., Welch M. (2013). Surface swarming motility by *Pectobacterium atrosepticum* is a latent phenotype that requires O antigen and is regulated by quorum sensing. Microbiology.

[32] Mastroeni P., Bryant C. (2004). Cytokines in Salmonellosis. EcoSal Plus.

[33] Nawab A., An L., Wu J., Li G., Liu W., Zhao Y., Wu Q., Xiao M. (2019). Chicken toll-like receptors and their significance in immune response and disease resistance. Int. Rev. Immunol..

[34] Rolin O., Muse S.J., Safi C., Elahi S., Gerdts V., Hittle L.E., Ernst R.K., Harvill E.T., Preston A. (2014). Enzymatic Modification of Lipid A by ArnT Protects *Bordetella bronchiseptica* against Cationic Peptides and Is Required for Transmission. Infect. Immun..

[35] Sivasankar C., Hewawaduge C., Lee J.H. (2023). Screening of lipid-A related genes and development of low-endotoxicity live-attenuated *Salmonella gallinarum* by arnT deletion that elicits immune responses and protection against fowl typhoid in chickens. Dev. Comp. Immunol..

[36] Giulietti A., Overbergh L., Valckx D., Decallonne B., Bouillon R., Mathieu C. (2001). An Overview of Real-Time Quantitative PCR: Applications to Quantify Cytokine Gene Expression. Methods.

[37] Lee S., Kwak J.-H., Kim S.H., Bin Jeong T., Son S.W., Kim J.-H., Lim Y., Cho J.-Y., Hwang D.Y., Kim K.S. (2019). Comparative study of liver injury induced by high-fat methionine- and choline-deficient diet in ICR mice originating from three different sources. Lab. Anim. Res..

[38] Doublet B., Douard G., Targant H., Meunier D., Madec J.-Y., Cloeckaert A. (2008). Antibiotic marker modifications of λ Red and FLP helper plasmids, pKD46 and pCP20, for inactivation of chromosomal genes using PCR products in multidrug-resistant strains. J. Microbiol. Methods.

[39] Wang Y., Berglund B., Zhu Y., Luo Q., Xiao Y. (2021). Performance of different methods for testing polymyxin B: Comparison of broth microdilution, agar dilution and MIC test strip in *mcr-1* positive and negative *Escherichia coli*. Lett. Appl. Microbiol..

[40] Behera B., Mathur P., Das A., Kapil A., Gupta B., Bhoi S., Farooque K., Sharma V., Misra M., Behera B. (2010). Evaluation of susceptibility testing methods for polymyxin. Int. J. Infect. Dis..

[41] Arunima A., Suar M. (2021). Glucose Starvation, Magnesium Ion Starvation, and Bile Stress Assays. Bio-Protocol.

[42] Neiger M.R., González J.F., Gonzalez-Escobedo G., Kuck H., White P., Gunn J.S. (2019). Pathoadaptive Alteration of *Salmonella* Biofilm Formation in Response to the Gallbladder Environment. J. Bacteriol..

[43] Hamadi F., Latrache H., Zahir H., Bengourram J., Kouider N., Elghmari A., Habbari K. (2011). Evaluation of the relative cell surface charge by using microbial adhesion to hydrocarbon. Microbiology.

[44] Livak K.J., Schmittgen T.D., Livak K.J., Schmittgen T.D. (2001). Analysis of Relative Gene Expression Data Using Real-Time Quantitative PCR and the 2^−ΔΔCT^ Method. Methods.

[45] Zou Y., Feng S., Xu C., Zhang B., Zhou S., Zhang L., He X., Li J., Yang Z., Liao M. (2012). The role of galU and galE of *Haemophilus parasuis* SC096 in serum resistance and biofilm formation. Vet. Microbiol..

[46] Guo L., Dai H., Feng S., Zhao Y. (2023). Contribution of GalU to biofilm formation, motility, antibiotic and serum resistance, and pathogenicity of *Salmonella* Typhimurium. Front. Cell. Infect. Microbiol..

[47] Abou-Shleib H., Elkhouly A., Roantree R.J. (1986). Effect of selected plasmids on the serum sensitivity of *Salmonella* Typhimurium strains with defined lipopolysaccharide core defects. FEMS Microbiol. Lett..

