# Supplementary material for: Deciphering the Interrelationship of arnT Involved in Lipid-A Alteration with the Virulence of Salmonella Typhimurium
Source: Int J Mol Sci. 2024 Feb 27;25(5):2760. doi: 10.3390/ijms25052760 (PMC10931828; doi:10.3390/ijms25052760)
Supplement: Supplementary file 1 [file ijms-25-02760-s001.zip › ijms-2821801-supplementary.pdf]

Supplementary Material

Deciphering the interrelationship of arnT involved in lipid-A alteration with the virulence of *Salmonella* Typhimurium

Chandran Sivasankar †, Khristine Kaith Sison Lloren † and John Hwa Lee *

College of Veterinary Medicine, Jeonbuk National University, Iksan 54596, Republic of Korea

* Correspondence: johnhlee@jbnu.ac.kr

† These authors contributed equally to this work.

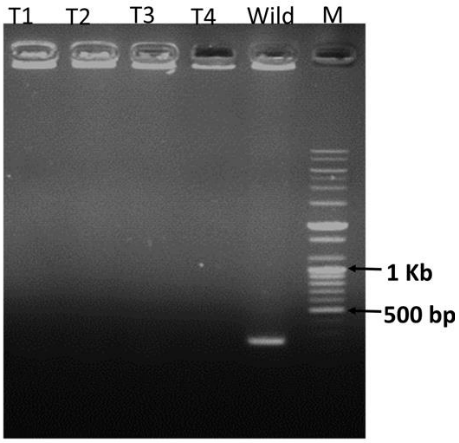

Figure S1. Inner primer PCR confirmation of *arnT* mutant with a band near 300bp only at wild type.

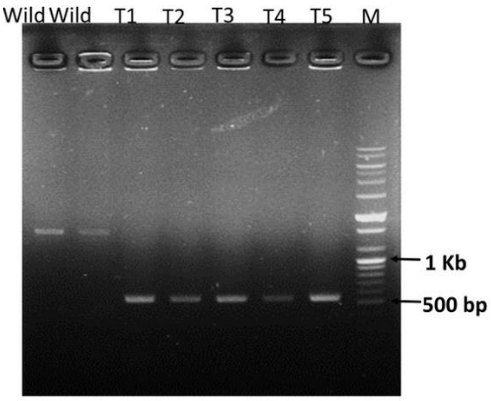

Figure S2. Flanking primer PCR confirmation of *arnT* mutant with a band at 1.6 kb in wild type and a band at 500 bp in *arnT* mutant.
